# Enrichment-free deep proteomics enables proteome-scale analysis of methionine oxidation

**DOI:** 10.1093/dnares/dsag011

**Published:** 2026-07-18

**Authors:** Hiromasa Mitsui, Yusei Okuda, Ryo Konno, Daisuke Nakajima, Osamu Ohara, Yusuke Kawashima

**Affiliations:** Department of Applied Genomics, Kazusa DNA Research Institute, Kisarazu, Chiba 292-0818, Japan; Department of Applied Genomics, Kazusa DNA Research Institute, Kisarazu, Chiba 292-0818, Japan; Department of Physics, School of Science, Kitasato University, Sagamihara, Kanagawa 252-0373, Japan; Department of Applied Genomics, Kazusa DNA Research Institute, Kisarazu, Chiba 292-0818, Japan; Department of Applied Genomics, Kazusa DNA Research Institute, Kisarazu, Chiba 292-0818, Japan; Department of Applied Genomics, Kazusa DNA Research Institute, Kisarazu, Chiba 292-0818, Japan; Department of Applied Genomics, Kazusa DNA Research Institute, Kisarazu, Chiba 292-0818, Japan; Department of Physics, School of Science, Kitasato University, Sagamihara, Kanagawa 252-0373, Japan

**Keywords:** methionine oxidation, methionine sulfoxide, oxidative stress, proteomics, enrichment-free analysis

## Abstract

Advances in mass spectrometry (MS)-based proteomics have enabled the large-scale characterization of posttranslational modifications (PTMs) through affinity-based enrichment. However, this technique introduces a bias towards selectively enrichable modifications, thus leaving oxidative modifications underexplored. Methionine oxidation (methionine sulfoxide) is an important indicator of cellular redox status, but its systematic analysis remains challenging because no enrichment method is available and artifactual oxidation can occur during sample preparation. Here, we developed an enrichment-free proteomic strategy for large-scale detection of methionine oxidation using a deep LC-MS platform. By optimizing acquisition conditions, we identified more than 260k precursors in a single-shot analysis. Under these conditions, methionine oxidation was efficiently detected, whereas many other PTMs remained poorly detected. To improve data reliability, we established a sample preparation workflow that minimized artifactual oxidation. Accordingly, we identified more than 3,500 methionine-oxidized proteins. Integration of methionine oxidation and expression proteomics across subcellular compartments revealed redox patterns under low-serum conditions, including increased mitochondrial oxidation and decreased endoplasmic reticulum oxidation. These changes are associated with metabolic reprogramming and altered antioxidant capacity. Overall, this study established an enrichment-free framework for the proteome-scale methionine oxidation analysis, and demonstrated that integrating oxidation and expression data enables the spatially resolved interpretation of cellular redox states.

## 1. Introduction

Recent advances in mass spectrometry (MS) instrumentation and analytical methodologies have established proteome analysis as a fundamental platform in life sciences.^1,2^ In particular, large-scale identification and quantification of post-translational modifications (PTMs) such as phosphorylation, ubiquitination, methylation, and glycosylation have been enabled by the development of affinity-based enrichment strategies using modification-specific antibodies or binding carriers.^3^ These technological advances have greatly improved our understanding of intracellular signaling pathways, protein functions, and disease-associated molecular networks. However, these successes depend largely on the availability of enrichment strategies for specific modifications. Consequently, proteomics suffers from an inherent technological bias toward analyzable modifications. Although PTMs amenable to selective enrichment have been extensively characterized, those lacking efficient enrichment methods remain poorly understood.^3,4^ Protein oxidation induced by reactive oxygen species (ROS) is implicated in a wide range of pathological conditions, including aging, cancer, metabolic disorders, and neurodegenerative diseases.^5,6^ Despite their broad biological significance, the global landscape of oxidative modifications remains insufficiently characterized, primarily due to the lack of robust and selective enrichment methods.

Methionine residues are among the most oxidation-prone amino acids in proteins and their oxidation state serves as an important indicator of the cellular redox environment and protein damage.^7,8^ Comprehensive analysis of methionine oxidation (methionine sulfoxide; MetO) would, therefore, enable sensitive and quantitative detection of oxidative stress at the molecular level. However, MetO induces only minimal changes in its isoelectric point and molecular mass, thereby making it difficult to generate antibodies capable of discriminating such subtle differences. Robust and broadly adopted antibody-based enrichment methods for MetO proteins/peptides have not yet been established, which limits the systematic investigation of this modification.^7^

Furthermore, MetO is not only a physiological modification but is also known to be artifactually induced during sample preparation in proteomic workflows. Such artifactual oxidation complicates the discrimination between endogenous and ex vivo modifications, and significantly compromises the reliability of quantitative analyses.^9,10^ Therefore, an accurate assessment of MetO requires advanced detection methodologies, along with the effective suppression of sample preparation-induced artifacts.

In this study, we developed an enrichment-free proteomic strategy based on a deep LC-MS system to enable direct and large-scale detection of MetO in proteins. In addition, we established an optimized sample preparation workflow that minimized artifactual MetO, thereby allowing reliable evaluation of endogenous oxidative modifications. Notably, this approach enables a large-scale quantitative assessment of MetO at the proteome level and provides a framework for the subcellular characterization of cellular oxidative states.

## 2. Materials and methods

### 2.1. Cell culture

HEK293T cells were cultured in Dulbecco’s Modified Eagle Medium (DMEM, high glucose) supplemented with 10% fetal bovine serum (FBS) at 37 °C in a humidified incubator with 5% CO_2_. For comparative analysis, cells were seeded at equal densities and cultured under either standard (10% FBS) or low-serum (2% FBS) conditions for 5 d prior to harvesting. Subsequently, the cells were detached using TrypLE Express (Thermo Fisher Scientific, Waltham, MA, USA) at 37 °C for 5 min and then collected in phosphate-buffered saline (PBS; Nacalai Tesque, Kyoto, Japan). Following collection, cells were precipitated at 500 × *g* and 4 °C for 2 min. The precipitate was frozen immediately at −80 °C and kept frozen until protein extraction. Cell numbers were recorded at the time of collection.

### 2.2. Protein extraction from HEK293T cells

Proteins from the HEK293T cells were extracted in protein extraction buffer (100 mM Tris-HCl (pH 8.0) and 20 mM NaCl containing 4% sodium dodecyl sulfate with or without 10 mM Methionine) by sonication in a Bioruptor II (CosmoBio, Tokyo, Japan) with settings at “High” and “30 s On/Off” cycle for a duration of 5 min. Additionally, protein concentration in the protein extract was determined using a ProteoAnalyzer (Agilent, Santa Clara, CA, USA) and adjusted to 100 ng/μL with protein extraction buffer with or without 10 mM Methionine.

### 2.3. Protein digestion

The 200 μL of protein lysate was subjected to clean up and digestion by the SP3-LASP method using Maelstrom 8 Autostage.^11^ Briefly, 2 types of SeraMag SpeedBead carboxylate-modified magnetic particles (hydrophilic particles: CAT# 45152105050250 and hydrophobic particles: CAT# 65152105050250; Cytiva, Marlborough, MA, USA) were used. The beads were combined in a 1:1 (v/v) ratio, washed twice with distilled water, and reconstituted in distilled water at a concentration of 10 μg solids/μL. Consequently, 20 μL of reconstituted beads was added to the sample followed by 1-propanol to bring the final concentration to 75% (v/v), with mixing for 5 min. The supernatant was discarded and the pellet was washed twice with 80% 1-propanol. Thereafter, the beads were resuspended in 80 μL of 50 mM Tris-HCl (pH 8.0), 10 mM CaCl_2_, and 0.02% lauryl maltose neopentyl glycol with or without 10 mM methionine, followed by addition of 1 μg of trypsin/Lys-C Mix (CAT# V5072, Promega, Madison, WI, USA), and mixed gently at 37 °C 16 h to digest proteins. The digested peptides were reduced and alkylated with 8 μL of 110 mM tris(2-carboxyethyl)phosphine and 440 mM 2-chloroacetamide at 80 °C for 15 min, followed by acidification with 16 μL of 5% TFA. The peptides were desalted using GL-Tip SDB (GL Sciences, Tokyo, Japan) according to the manufacturer’s protocol, eluted with 34% acetonitrile in 0.1% TFA, and dried using a centrifugal evaporator (miVac Duo Concentrator; Genevac, Ipswich, UK). Subsequently, the dried peptides were reconstituted in 0.02% DMNG containing 0.1% TFA, and concentrations were determined using a Pierce Quantitative Fluorescent Peptide Assay kit (Thermo Fisher Scientific) and adjusted to 500 ng/µL with the same solvent.

### 2.4. NanoLC–MS/MS

Nano-LC–MS/MS analyses were performed using a Vanquish Neo UHPLC system (Thermo Fisher Scientific) with 3 different LC programs: 15 SPD, 20 SPD, and 30 SPD. The redissolved peptides were directly injected onto a 75 µm × 30 cm nanoLC column (ReproSil-Pur C18, 1.5 µm particle size, 100 Å pore size; CoAnn Technologies, Richland, WA, USA) maintained at 60 °C without using a trap column. Mobile phase A consisted of 0.1% formic acid in distilled water and mobile phase B consisted of 0.1% formic acid in 80% acetonitrile. For the 15 SPD method, peptides were separated using an 84.5-min gradient as follows: 1% B at a flow rate of 600 nL/min for 0 to 0.5 min; 1 to 8% B at 600 to 200 nL/min for 0.5 to 4.5 min; 8 to 24% B at 200 nL/min for 4.5 to 60 min; 24 to 40% B at 200 nL/min for 60 to 78.5 min; 40 to 98% B at 200 nL/min for 78.5 to 79.5 min; 98% B at 200 nL/min for 79.5 to 80.5 min; 98% B at 200 to 600 nL/min for 80.5 to 81.5 min; 98% B at 600 to 750 nL/min for 81.5 to 82.5 min; and 98% B at 750 nL/min for 82.5 to 84.5 min. Further, for the 20 SPD method, peptides were separated using a 60.5-min gradient as follows: 1% B at a flow rate of 600 nL/min for 0 to 0.5 min; 1 to 8% B at 600 to 200 nL/min for 0.5 to 4.5 min; 8 to 24% B at 200 nL/min for 4.5 to 42 min; 24 to 40% B at 200 nL/min for 42 to 54.5 min; 40 to 98% B at 200 nL/min for 54.5 to 55.5 min; 98% B at 200 nL/min for 55.5 to 56.5 min; 98% B at 200 to 600 nL/min for 56.5 to 57.5 min; 98% B at 600 to 750 nL/min for 57.5 to 58.5 min; and 98% B at 750 nL/min for 58.5 to 60.5 min. For the 30 SPD method, peptides were separated using a 36.5-min gradient as follows: 1% B at a flow rate of 600 nL/min for 0 to 0.5 min; 1 to 8% B at 600 to 300 nL/min for 0.5 to 3.5 min; 8 to 24% B at 300 nL/min for 3.5 to 24.5 min; 24 to 40% B at 300 nL/min for 24.5 to 31.5 min; 40 to 98% B at 300 nL/min for 31.5 to 32.5 min; 98% B at 300 nL/min for 32.5 to 33.5 min; 98% B at 300 to 600 nL/min for 33.5 to 34.5 min; 98% B at 600 to 750 nL/min for 34.5 to 35 min; and 98% B at 750 nL/min for 35 to 36.5 min. At the beginning of each gradient, 1% mobile phase B was delivered at a high flow rate during sample loading, which allowed column equilibration to occur during the loading step and eliminated the need for an additional post-run equilibration step. Importantly, this strategy shortened the total LC cycle time and improved sample throughput. At the end of each gradient, 98% mobile phase B was delivered at a high flow rate to efficiently wash the analytical column and reduce carryover.

Moreover, the eluted peptides were analyzed using an Orbitrap Astral mass spectrometer (Thermo Fisher Scientific) equipped with an InSpIon system.^12^ MS1 spectra were acquired over an *m/z* range of 380 to 980 at a resolution of 240,000 using an Orbitrap analyzer with an automatic gain control (AGC) target of 300% and a maximum injection time of 5 ms. MS2 spectra were acquired over an *m/z* range of 200 to 2,000 using an Orbitrap Astral analyzer with an AGC target of 600%, a maximum injection time of 3.5 ms, and a normalized collision energy of 25%. Additionally, the isolation width for MS2 was set to 2 Th with window placement optimization.

### 2.5. Protein identification and quantitative analysis from MS data

DIA raw files were processed in Spectronaut v20.4 (Biognosys, Schlieren, Switzerland) using the directDIA + workflow. Briefly, raw files were searched against the human protein sequence UniProt database (proteome ID UP000005640; 20,656 entries downloaded on 1 November 2024). Search parameters were set as follows: enzyme, Trypsin/P; maximum missed cleavages, 2; peptide length, 7 to 45; precursor charge, 2 to 4; maximum variable modifications, 3; fixed modification, “Carbamidomethylation (C)”; variable modifications, “Acetyl (Protein N-term),” and “Oxidation (M)”; and MS1 and MS2 mass tolerances, 10 ppm. Identification was filtered at 1% false discovery rate (FDR) at the PSM, precursor, and protein levels. Further, PTM localization was enabled, and the site confidence score cutoff was set to 0.75.

For PTM-specific analyses, each search was conducted independently in Spectronaut with Acetyl (Protein N-term) included as a common variable modification, and exactly one target PTM included per search. The examined PTMs were Phospho (STY), Acetyl (K), Methyl (K), Dimethyl (KR), Trimethyl (K), GlyGly (K), Oxidation (M), and Deamidation (NQ). Quantification and normalization were performed in Spectronaut. Accordingly, protein group quantities were calculated from peptide quantities using the Top3 approach, and peptide quantities were calculated from precursor quantities using the Top3 approach. For both protein and peptide analyses, the resulting quantitative values were normalized across samples using local regression normalization in Spectronaut.

### 2.6. Data analysis

Protein intensities and MetO peptide intensities were log2-transformed, and data were filtered to retain proteins with at least 70% valid values in at least one group. Missing values were imputed using random numbers drawn from a normal distribution (width = 0.3, downshift = 1.8) in Perseus v1.6.15.012. Thereafter, differentially expressed proteins were defined as those displaying more than a 2-fold change with *P* < 0.05 (Student’s *t*-test) between the two groups, and volcano plots were generated based on these criteria. Additionally, methionine-oxidized peptides derived from proteins specific to each subcellular localization category were summed for each localization category. Information on subcellular localization-specific proteins was obtained from the Human Protein Atlas (https://www.proteinatlas.org/humanproteome/subcellular).

## 3. Results

### 3.1. Optimization of deep proteome coverage and detection characteristics of post-translational modifications

**Fig. 1.**
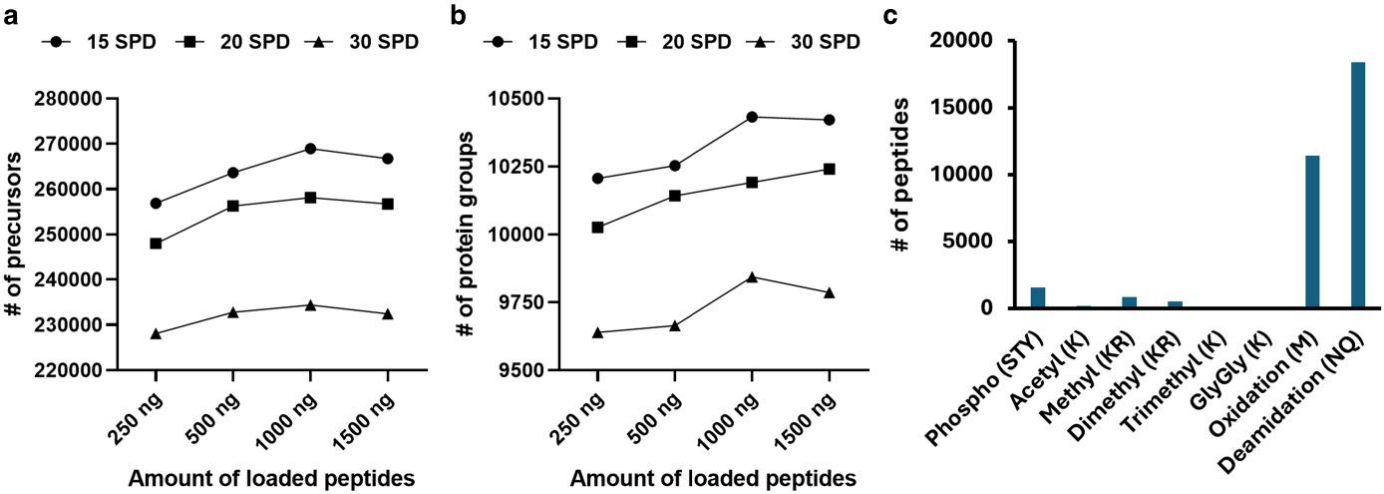
Optimization of Astral analytical conditions and feasibility of PTM analysis without enrichment. (a, b) Precursors and protein groups identifications were evaluated using Astral across analytical methods with different run times and peptide loading amounts. Run times corresponding to 15, 20, and 30 samples per day (SPD) were tested, with peptide loads of 250, 500, 1,000, and 1,500 ng. (c) Various PTM analyses were conducted using MS data acquired at 15 SPD with a 1,000 ng peptide load. Each PTM was analyzed individually using Spectronaut.

To achieve deep proteome coverage without enrichment, we employed a high-performance mass spectrometer (Orbitrap Astral) and optimized analytical parameters, including gradient length and peptide loading (Fig. 1a, b). We used HEK293T cells as the model system and aimed to identify more than 260,000 precursors in a single-shot analysis. Under optimized conditions, a 15 samples-per-day (SPD) method enabled the identification of more than 260,000 precursors with peptide loading amounts ranging from 500 to 1,500 ng. Among these conditions, a loading amount of 1,000 ng resulted in the highest number of identified proteins, peptides, and precursors.

Next, we evaluated the detectability of post-translational modifications using this deep proteome dataset (Fig. 1c). Rather than performing a combined search of multiple modifications, each modification type was independently analyzed using Spectronaut. Each PTM was analyzed separately to evaluate which PTMs tended to be detected more frequently under the present enrichment-free deep proteomics conditions. This strategy avoided excessive expansion of the search space caused by simultaneous searching of multiple variable modifications, which may reduce identification sensitivity and increase the risk of false identifications. Notably, since multiple independently searched PTM datasets were integrated, the comparison should be interpreted with caution as an exploratory assessment of modification detectability rather than as a direct comparison of absolute PTM abundance. Consequently, representative modifications such as phosphorylation, ubiquitination (GlyGly), acetylation, and methylation remained poorly detected even under deep proteome coverage conditions. In contrast, high levels of MetO and asparagine/glutamine deamidation were detected, with more than 10,000 modified peptides identified for each modification. Although these modifications may have been partially introduced during sample preparation, their high detectability is likely attributable to the fact that they do not substantially alter the peptide charge states. These findings suggest that modifications with a minimal impact on peptide ionization properties are preferentially detected in enrichment-free proteomics.

### 3.2. Optimization of sample preparation to suppress artifactual MetO

**Fig. 2.**
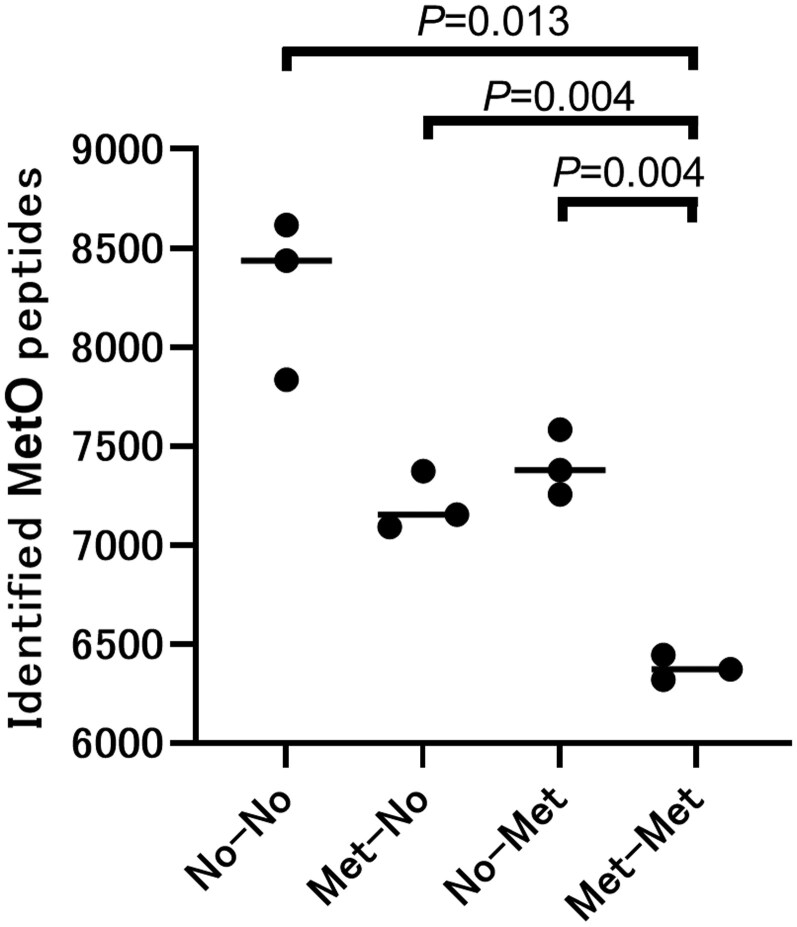
Effects of adding 10 mM methionine to the protein extraction solution and/or the digestion solution to prevent artifactual methionine oxidation during sample preparation. The number of methionine-oxidized (MetO) peptides identified under each condition was used to evaluate suppression of artifactual methionine oxidation (*n* = 3). “No–No” indicates that methionine was not added to either the protein extraction solution or the digestion solution. “Met–No” indicates that methionine was added to the protein extraction solution but not to the digestion solution. “No–Met” indicates that methionine was not added to the protein extraction solution but was added to the digestion solution. “Met–Met” indicates that methionine was added to both the protein extraction and digestion solutions.

Based on the above findings, we sought to optimize the sample preparation conditions to suppress artifactual MetO. Accordingly, we employed a 30 SPD acquisition method to maximize detection sensitivity during condition screening. We evaluated the effect of methionine supplementation in both the protein extraction buffer and the digestion solution (Fig. 2). Although higher methionine concentrations may further increase the scavenging capacity against oxidation, concentrations above 10 mM were avoided based on preliminary observations during our method optimization, since they could affect tryptic digestion efficiency and interfere with subsequent peptide quantification. Therefore, 10 mM was chosen as an optimal concentration that provided effective suppression of artifactual oxidation, while minimizing potential effects on sample preparation and LC–MS analysis.

**Fig. 3.**
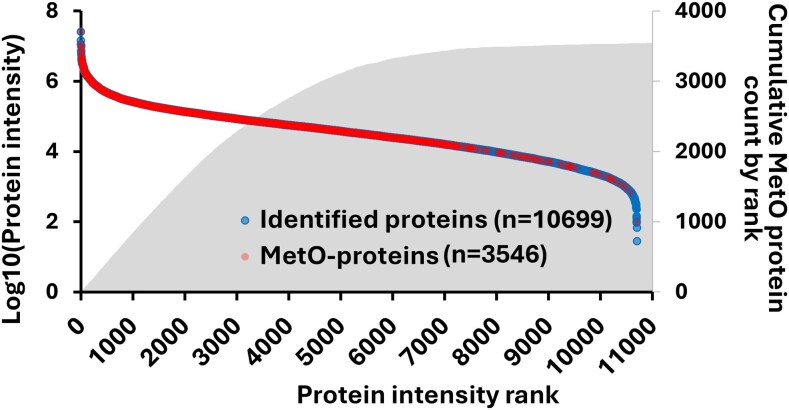
Dynamic range of protein intensities in HEK293T samples with suppressed methionine oxidation during sample preparation, measured using the astral 15SPD method. Blue dots represent the identified proteins, while red dots indicate proteins containing methionine oxidation. The gray area represents the cumulative number of MetO peptides ranked by protein intensity.

The addition of methionine at either step resulted in a reduction in MetO levels. Notably, supplementation in both the extraction and digestion steps led to a more pronounced suppression than either condition alone. Based on these results, we adopted a protocol that included methionine supplementation during both the extraction and digestion steps for subsequent analyses. Using this optimized workflow, HEK293T-derived peptides were analyzed under 15 SPD conditions, which resulted in the identification of 3,546 MetO proteins in a single-shot analysis (Fig. 3). However, the identified MetO proteins were predominantly derived from highly abundant proteins. This indicates that, while large-scale analysis is achievable without enrichment, the detection of oxidation events in low-abundance proteins remains limited.

### 3.3. Integration of subcellular annotation-based MetO and expression proteomics reveals localization-associated redox alterations

**Fig. 4.**
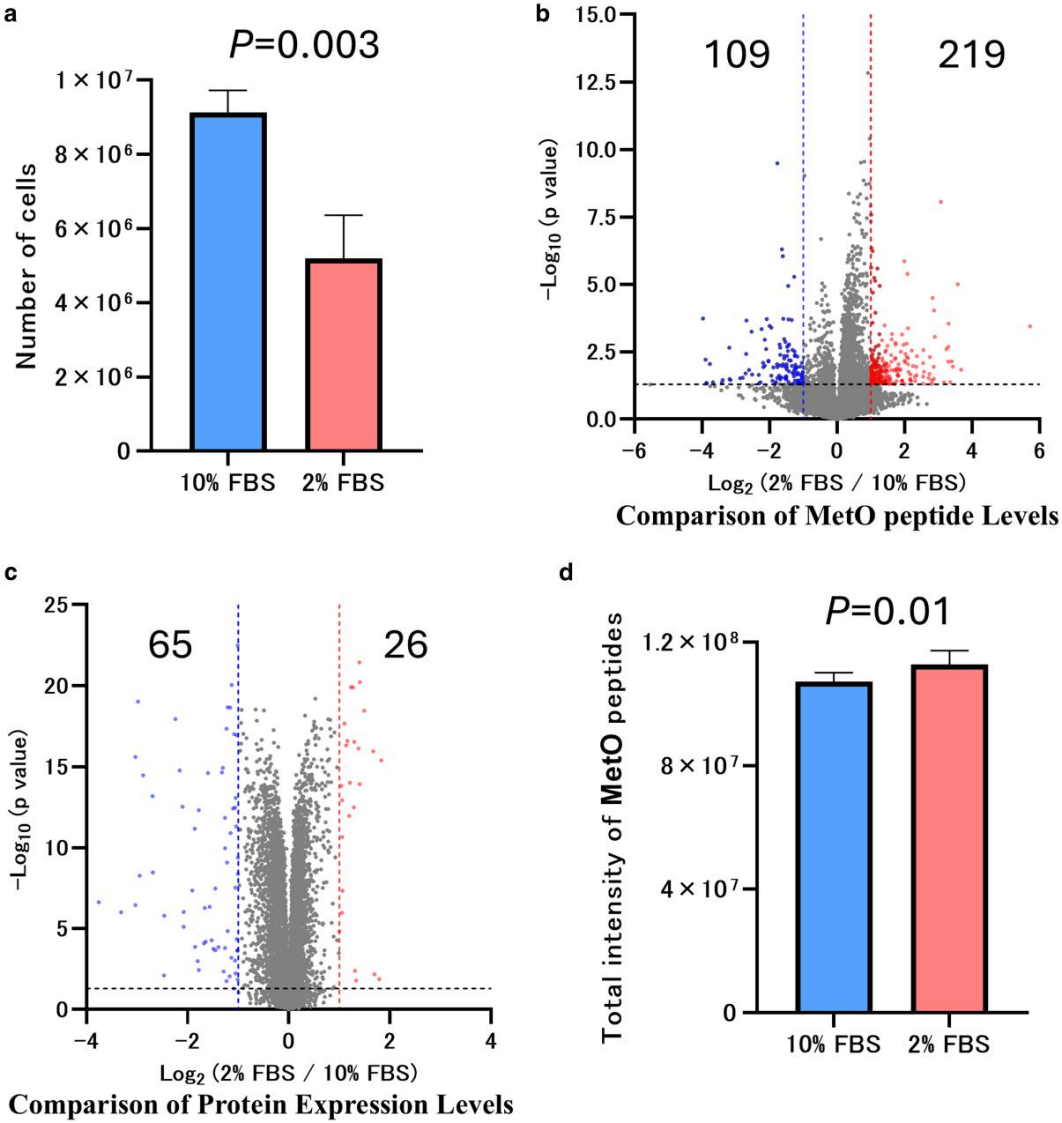
Effects of low-serum conditions on cell growth, methionine oxidation, and protein expression. a) Comparison of cell numbers at harvest when the same number of cells were seeded at passage and cultured under 10% FBS and 2% FBS conditions (*n* = 8 each). b) Volcano plot of methionine-oxidized peptide intensities comparing the 10% and 2% FBS conditions (*n* = 8 each). c) Volcano plot of protein expression levels comparing the 10% and 2% FBS conditions (*n* = 8 each). d) Comparison of total methionine-oxidized peptide intensities between the 10% and 2% FBS conditions (*n* = 8 each).

To investigate redox regulation at the subcellular level, we integrated MetO profiles with expression proteomics using the same samples. Therefore, HEK293T cells cultured in 10% and 2% FBS were comparatively analyzed. The 2% FBS condition was designated as a low-serum/starvation-like condition that maintains sufficient cell viability while reducing serum-derived growth factor and nutrient signaling. Since serum deprivation can induce metabolic adaptation and redox-related stress responses, including altered mitochondrial function and ROS production, this condition was considered suitable for evaluating changes in cellular redox status and MetO levels without causing extensive cell death. Despite equal seeding densities, cell numbers after 5 d were approximately 9 × 10^6^ under 10% FBS and 5 × 10^6^ under 2% FBS, thereby indicating reduced proliferation under low-serum conditions (Fig. 4a).

**Fig. 5.**
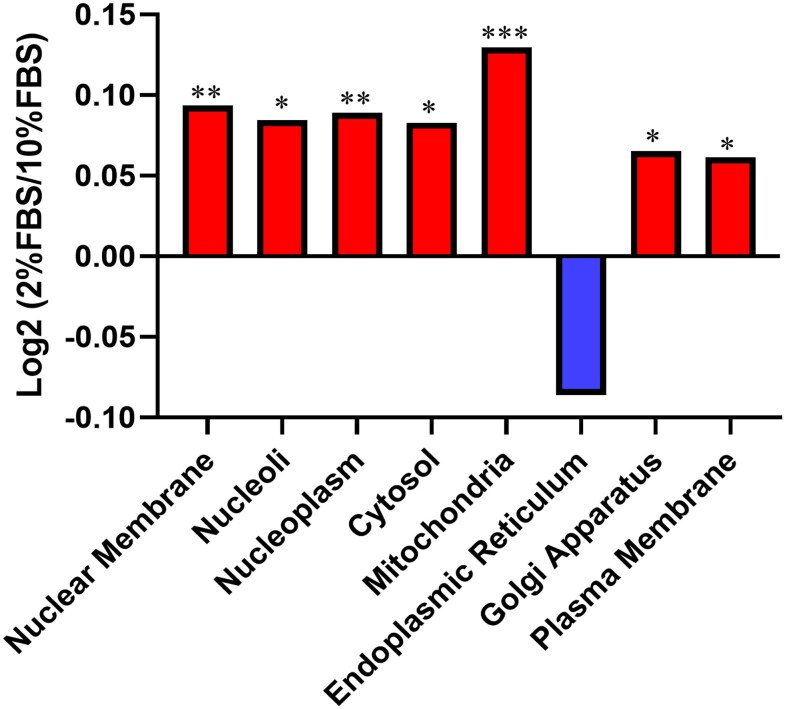
Comparison of methionine oxidation levels across subcellular localizations. Methionine-oxidized peptides derived from proteins specific to each subcellular localization category were summed. The *y*-axis represents the log2-transformed ratio of 2% FBS to 10% FBS for each localization. Subcellular localizations that increased in 2% FBS are shown in red, while those that decreased are shown in blue. Statistical significance between the 2 groups (*n* = 8 each) was assessed and is indicated by *P < 0.05, **P < 0.01, and ***P < 0.001.

**Fig. 6.**
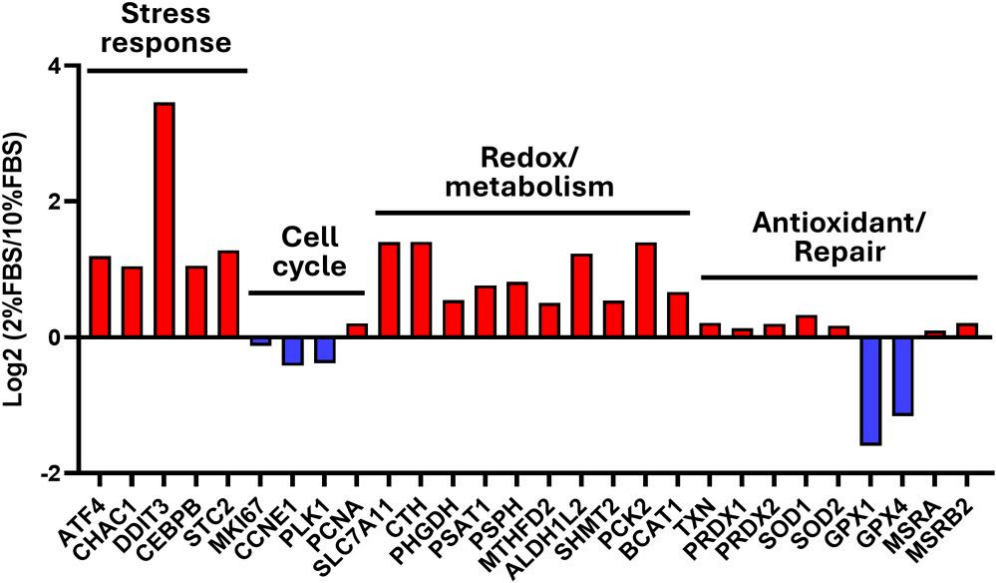
Comparison of expression levels of proteins associated with biological processes affected by low-serum conditions and proteins involved in oxidative processes. The *y*-axis represents the log2-transformed ratio of protein intensity (2% FBS/10% FBS) for each identified protein (*n* = 8 each). Proteins that increased in 2% FBS are shown in red, whereas those that decreased are shown in blue. All comparisons between the two groups were statistically significant (*P* < 0.001).

Further, under low-serum conditions, a larger number of proteins displayed decreased expression levels, whereas more peptides exhibited increased abundance at the MetO peptide level (Fig. 4b, c). Consistent with this observation, the overall levels of MetO peptides underwent a modest increase under low-serum conditions (1.04-fold, *P* = 0.02; Fig. 4d). However, the subcellular analysis revealed distinct compartment-dependent patterns (Fig. 5). The most prominent increase was observed in mitochondria (1.09-fold, *P* = 7.0 × 10^−4^), while moderate increases were observed in nuclear and cytosolic compartments (∼1.06-fold, *P* < 0.05). In contrast, the endoplasmic reticulum was the only organelle that displayed a downward trend (0.94-fold), although this change was not statistically significant. To interpret these compartment-specific differences, we integrated the expression proteomic data (Fig. 6, Table S1). Under 2% FBS conditions, stress-response-related proteins, including ATF4, CHAC1, DDIT3, CEBPB, and STC2, were significantly upregulated, which indicated the activation of adaptive stress responses.^13^ In parallel, cell cycle–related proteins, such as MKI67, CCNE1, and PLK1, demonstrated modest decreases, which was consistent with the reduced proliferation observed at the cellular level. Notably, PCNA did not display a decrease comparable to that of other cell cycle–related proteins. Given its role in DNA replication and repair, this observation may be consistent with the activation of replication stress-related responses under low-serum conditions.^14^ Marked changes were also observed in the proteins involved in redox homeostasis and metabolism. Amino acid metabolism and redox-related pathways were upregulated, including SLC7A11, CTH, enzymes involved in serine biosynthesis (PHGDH, PSAT1, and PSPH), and those involved in one-carbon metabolism (MTHFD2 and ALDH1L2). Additionally, multiple antioxidant systems, including TXN, PRDX family proteins, and SOD1/2 exhibited increased expression. In contrast, GPX1 and GPX4 were markedly downregulated (0.3-fold and 0.4-fold, respectively; *P* < 10^−14^), thereby suggesting a potential reduction in peroxide detoxification capacity. Further, methionine sulfoxide reductases (MSRA and MSRB2) displayed modest upregulation, which suggests adaptive modulation of repair systems. Collectively, these patterns suggest that the antioxidant and repair systems may be differentially regulated under low-serum conditions.

At the individual protein level, methionine oxidation increased in functionally distinct clusters (Table S2). Notably, mitochondrial proteins involved in metabolism and quality control, including PCK2, ALDH1L2, TRAP1, LONP1, OPA1, NDUFS2, and COQ8A, were prominently represented, which is consistent with the elevated mitochondrial oxidative burden. Furthermore, proteins involved in serine biosynthesis, one-carbon metabolism, and transsulfuration pathways, such as PHGDH, PSAT1, PSPH, SHMT2, CTH, BCAT1, and SLC7A11, exhibited increased MetO levels. These proteins were simultaneously upregulated at the expression level, thus raising the possibility that the metabolic pathways involved in redox adaptation are subject to oxidative modifications. In contrast, although the endoplasmic reticulum showed an overall decreasing trend in MetO, selected ER and secretory pathway proteins, including HSP90B1, TMED2, TMED9, POFUT1, and CLCC1, exhibited increased oxidation. These findings suggest that redox changes in the ER are not uniform but occur in a function-specific manner.

Collectively, these results indicate that redox changes under low-serum conditions are not uniform across the cell, but involve compartment-dependent alterations characterized by an increased oxidative burden in mitochondria and a possible reduction in functional demand in the endoplasmic reticulum. The integration of MetO and expression proteomics provides a framework for understanding redox remodeling across cellular compartments and functional networks.

## 4. Discussion

In this study, we developed an enrichment-free deep proteomics strategy that enables large-scale detection of MetO, and integrated it with expression proteomics to investigate redox regulation at the subcellular level. Our results demonstrate that MetO can be detected at a scale without enrichment, and that its integration with expression data provides a framework for interpreting cellular redox states in a compartment-dependent manner. The preferential detection of MetO in our enrichment-free deep LC-MS workflow may reflect not only the relatively modest effect of methionine oxidation on peptide ionization, but also other analytical factors inherent to enrichment-free PTM detection. These include modification stoichiometry, absence of PTM-specific enrichment, peptide abundance, and fragmentation-method-dependent identification efficiency. The identification of 10,699 MetO peptides from 3,546 proteins highlights the depth of the developed workflow and places it among the largest MetO proteomic datasets reported to date, particularly among studies performed without dedicated enrichment of MetO peptides. For comparison, a COFRADIC-based Jurkat cell study identified 2,626 MetO-containing peptides in 1,655 proteins,^8^ and an Arabidopsis oxidative stress study reported over 500 oxidation sites in approximately 400 proteins,^15^ whereas a human proteome-wide MetO study using oxidation rates as a structural probe covered approximately 3,000 proteins rather than direct large-scale profiling of endogenous MetO proteins.^16^

A key finding of this study was that MetO exhibited distinct subcellular patterns under low-serum conditions. While global changes were modest, subcellular analysis revealed a pronounced increase in mitochondrial oxidation, which was accompanied by moderate increases in the nuclear and cytosolic compartments. In contrast, the endoplasmic reticulum exhibited a decreasing trend, thereby suggesting that redox changes were not uniformly distributed across the cell. These observations indicate that MetO captures spatial heterogeneity in cellular redox states that cannot be resolved by bulk measurements alone.

Integration with expression proteomics has further enabled the functional interpretation of these compartment-specific changes. The upregulation of proteins associated with the integrated stress response (ISR), including ATF4, DDIT3, and CHAC1, indicated the activation of adaptive stress responses under low-serum conditions.^13^ Concurrently, the metabolic pathways associated with redox homeostasis, including serine biosynthesis, one-carbon metabolism, and transsulfuration, were upregulated. These pathways are closely linked to NADPH production and redox buffering capacity, which suggests that the observed proteomic changes reflect metabolic reprogramming associated with adaptation to nutrient limitation and oxidative stress.^17^ Simultaneously, cell cycle–related proteins displayed only modest decreases despite a clear reduction in cell proliferation. This discrepancy highlights the importance of integrating the cellular and proteomic measurements. Notably, PCNA expression did not follow the trend observed for other cell cycle proteins, which may reflect its additional role in DNA replication and repair.^14^ This observation suggests that replication-associated stress responses remain active, even under reduced proliferation conditions. Notably, this paper revealed the differential regulation of the antioxidant and repair systems. Although GPX1 and GPX4—key enzymes responsible for peroxide detoxification—were strongly downregulated, methionine sulfoxide reductases (MSRA and MSRB2) were modestly upregulated. This opposing regulation suggests that redox control under low-serum conditions is not uniformly enhanced, but rather reconfigured. Specifically, our data are consistent with a model in which the peroxide detoxification capacity is reduced, leading to increased accumulation of oxidative modifications, while repair systems are activated to compensate for this increased oxidative burden. This shift might reflect an adaptive strategy under nutrient-limited conditions. Additionally, antioxidant systems such as the glutathione-dependent GPX pathway require reducing equivalents, including NADPH, which can become limiting under low-serum conditions. In contrast, repair mechanisms that target oxidized residues may represent localized and resource-efficient strategies for maintaining protein function. Thus, cells may shift toward a state resembling a “prevention” strategy, wherein oxidative damage is suppressed, to a “tolerance and repair” strategy, in which damage is permitted but subsequently corrected.

At the individual protein level, MetO was enriched in functionally coherent groups, particularly in mitochondrial proteins involved in metabolism and quality control. These findings are consistent with increased oxidative pressure in mitochondria, which is likely associated with metabolic reprogramming. Furthermore, proteins involved in amino acid metabolism and redox-related pathways exhibited coordinated increases in both expression and MetO. This suggests that the pathways activated in response to stress are subject to oxidative modifications, thereby indicating a complex interplay between metabolic activation and oxidative stress. Conversely, the endoplasmic reticulum exhibited an overall decreasing trend in the MetO group despite the increased oxidation of selected proteins. Given that the ER is a major site for oxidative protein folding, this observation may reflect, at least in part, a reduction in protein-folding demand, rather than an enhanced antioxidant capacity.^18^ This interpretation is consistent with reduced proliferation and suggests that redox changes in the ER are driven by functional load rather than oxidative stress.

Importantly, recent advances in MS have enabled sufficient proteome coverage in single-shot analyses,^2,19,20^ thereby shifting the focus of proteomics from increasing analytical depth to improving throughput.^21–23^ However, our results highlighted that analytical depth remains a critical factor in detecting post-translational modifications without enrichment. In particular, modifications such as MetO, which do not benefit from affinity-based enrichment strategies, require deep proteome coverage for comprehensive characterization. In this study, DIA data were acquired using an Orbitrap Astral mass spectrometer with a narrow isolation window of 2 Th, which is comparable to the isolation width commonly used in conventional DDA acquisition. Recent Orbitrap Astral-based studies have demonstrated that DIA can provide broader coverage of modified peptides than DDA-based workflows, thereby reaffirming the use of DIA for large-scale PTM peptide detection.^24^ Moreover, these findings support the use of DIA data for evaluating MetO peptide detection and quantification in the present enrichment-free workflow. In addition to the acquisition strategy, sufficient LC separation depth was important for detecting low-abundance MetO peptides without enrichment. Therefore, we used a relatively long 84.5-min LC gradient in the optimized workflow. Under this condition, 1,000 ng peptide loading improved MetO peptide detection without substantial deterioration of chromatographic peak shape or unacceptable elevation of column pressure. Thus, this loading amount was suitable for the present enrichment-free, high-depth MetO workflow. However, the optimal loading amount may vary depending on the LC system, analytical column, gradient length, sample complexity, and analytical purpose, and therefore, should be optimized for each analytical platform and workflow. Further, MetO peptide intensity should be interpreted as observed MetO peptide abundance rather than absolute oxidation stoichiometry or site occupancy. Although changes in parental protein abundance may influence MetO peptide intensity, simple normalization by parental protein abundance does not directly estimate methionine oxidation occupancy because it is not based on the ratio between oxidized and unmodified forms of the same peptide. Moreover, ROS are finite, short-lived, and consumed by numerous competing oxidation targets and antioxidant systems. Accordingly, ROS-mediated oxidation does not necessarily scale linearly with the abundance of a single parental protein. Therefore, MetO peptide intensity and summed MetO peptide intensity were retained as primary readouts, and the corresponding parental protein abundances are provided in Table S3 to aid interpretation.

Collectively, these findings demonstrate that MetO is not merely a marker of oxidative damage, but may also provide functional insight into cellular redox regulation when analyzed in a spatial and integrative context. By combining subcellular MetO with expression proteomics, this study established a framework for dissecting redox alterations associated with subcellular localization.

Despite its strengths, this study also had a few limitations. First, although the addition of free methionine reduced the artifactual methionine oxidation during sample preparation, the present workflow does not completely distinguish pre-existing endogenous MetO from oxidation newly introduced during sample handling; this would require isotope-based strategies such as ^18^O-labeled oxidation/blocking approaches. However, this workflow was designed for comparative MetO profiling across samples processed under identical conditions, rather than for absolute separation or quantification of endogenous and sample preparation-induced MetO in individual samples. This design ensures that sample preparation–derived oxidation artifacts are uniformly distributed across groups, while free methionine supplementation minimizes background MetO signals, thereby enhancing detection of biologically meaningful differences in endogenous MetO.

Second, the subcellular analysis was based on localization annotations from the Human Protein Atlas rather than experimental organelle fractionation. Based on these annotations, we observed differential changes in protein abundance and MetO levels among proteins associated with specific organelles, particularly those involved in redox regulation and cellular homeostasis. Although these results do not directly demonstrate compartment-dependent redox remodeling, they suggest that redox-related alterations may occur in a localization-associated manner. Third, the analysis was based on protein abundance and peptide-level oxidation measurements, and did not directly assess enzyme activity or local ROS levels. Fourth, the stereochemistry of methionine sulfoxide and specificity of methionine sulfoxide reductases were not evaluated. Therefore, future studies integrating isotope-based validation, organelle fractionation, activity assays, metabolic flux analyses, and real-time ROS measurements are expected to further refine our understanding of redox regulation.

In conclusion, our enrichment-free deep proteomics approach enables the systematic analysis of MetO and reveals subcellular localization-associated redox alterations under low-serum conditions. Importantly, this framework provides a foundation for investigating redox regulation in diverse biological contexts, including disease, metabolic stress, and aging.

## Supplementary Material

dsag011_Supplementary_Data
